# Factors associated with viral load non-suppression among adults with HIV in Sughd region, Tajikistan: a retrospective cohort study

**DOI:** 10.1186/s12879-025-11270-1

**Published:** 2025-07-07

**Authors:** Emomali Qurbonov, Dilyara Nabirova, Aisuluu Kubatova, Salomudin Yusufi, Edmond F. Maes, Roberta Horth

**Affiliations:** 1 Department of Epidemiology, Sughd Regional Center for Prevention and Control of HIV, Khujand, Tajikistan; 2 Central Asia Field Epidemiology Training Program, Asfendiyarov Kazakh National Medical University, Almaty, Kazakhstan; 3 Division of Global Health Protection in Central Asia, United States Centers for Disease Control and Prevention, Almaty, Kazakhstan; 4 National Institute of Public Health, Ministry of Health of the Kyrgyz Republic, Bishkek, Kyrgyzstan; 5 Science Department, Ministry of Health and Social Protection of the Population of the Republic of Tajikistan, Dushanbe, Tajikistan; 6 Department of Global Health, Emory Rollins School of Public Health, Georgia Atlanta, USA

**Keywords:** Adults, Retrospective studies, Viral load, Tajikistan, Migrants, HIV infections

## Abstract

**Background:**

Viral load suppression among people living with HIV is a key strategy for reducing HIV transmission. A global target for HIV elimination aims to have 95% of people living with HIV diagnosed, 95% of people diagnosed on antiretroviral therapy (ART), and 95% viral load suppression for those on ART. We aimed to assess viral load non-suppression rates and associated factors among people living with HIV on ART in the Sughd region of Tajikistan.

**Methods:**

We conducted a retrospective cohort study of adults (≥ 18 years old) who were newly diagnosed with HIV in 2013–2022 and had received ART for ≥ 6 months in the Sughd Region. Data were collected from the national electronic HIV case surveillance system and cross-referenced with paper medical and laboratory records. We conducted multivariable Quasi-Poisson regression to identify factors associated with viral load non-suppression (defined as ≥ 1000 copies/mL on their latest viral load test).

**Results:**

Among the 1,871 people newly diagnosed with HIV who received ART for ≥ 6 months from 2013 to 2022, 11% were not virally suppressed. Over half (57%) were male, 38% were migrants, 73% were married, and the median age was 31 years (range 18–74). One-third (32%) had advanced HIV disease at diagnosis, 58% had been on ART for < 5 years, 94% were on a dolutegravir-containing regimen (DTG), and 9% died. Viral load non-suppression was 23% among people with stage IV at diagnosis and 43% among those not on DTG. Higher risk of viral load non-suppression was observed among male migrants and male nonmigrants compared to female nonmigrants (adjusted relative risk [aRR] and 95% confidence interval = 1.61 [1.13–2.31] and aRR = 1.48 [1.03–2.14], respectively), those who never-married vs. married (aRR = 1.56 [1.05–2.25]), those on ART for < 5 years vs. longer (aRR = 1.56 [1.05–2.29]), those initiating ART in 2013–2018 compared to 2019–2020 (aRR = 1.92 [1.28–2.88]), and those not on DTG (aRR = 3.86 [2.63–5.69]).

**Conclusions:**

Viral load suppression among people living with HIV in the Sughd Region remains below the global 95% target. Viral load suppression may improve with increased treatment support for people with late diagnosis or those newly initiating ART, with a special focus on men and migrants.

## Background

Substantial progress has been achieved in reducing new HIV infections globally over the last two decades, yet HIV remains a significant public health challenge [1–3]. There were 39.9 million people living with HIV globally in 2023, including 1.3 million incident HIV infections and 630,000 people who died from HIV-related illnesses [4]. There is still no cure for HIV, and treatment is a lifelong process that requires daily antiretroviral therapy (ART) and regular healthcare follow-ups [5].

When taken as prescribed, ART suppresses the amount of HIV in the body to undetectable levels [6]. People living with HIV who are virally suppressed have reduced mortality and improved quality of life [7]. Viral load suppression also eliminates sexual transmission of HIV to partners [8, 9], making it a critical goal for reducing HIV incidence at a global level [10–12].

Identification of specific barriers to viral suppression is necessary for countries to reach HIV elimination targets, in which 95% of people living with HIV know their status, 95% of those diagnosed with HIV are on ART, and 95% of those on ART achieve viral suppression [13]. Studies have found that viral load suppression can be improved by rapid ART initiation, regular viral load testing, and support with treatment adherence [14–17].

In Tajikistan, through expanded HIV testing and treatment, the number of people diagnosed with HIV and on ART has increased over the last decade [18]. However, the proportion of people who know they have HIV, are on ART, and achieve viral suppression remains below target. As of 2023, out of an estimated 15,000 people living with HIV in Tajikistan, just 10,000 (67%) were on ART, and 9,300 (or 62%) were virally suppressed. Challenges to achieving epidemic control in the country include high mobility, stigma and discrimination, and service delivery challenges [19].

We aimed to identify factors associated with viral load non-suppression among adults living with HIV who have been receiving antiretroviral therapy for more than 6 months in the Sughd Region. The Sughd Region is the second-most populous region in the country, where 2,371 people living with HIV were receiving antiretroviral therapy in 2023 [20]. The findings of this study can provide information that national and local health authorities can use in their efforts to reach epidemic control in Tajikistan.

## Methods

### Study design

We conducted a retrospective cohort study among people aged 18 years and older who had been newly diagnosed with HIV from January 1, 2013 to December 31, 2022, and who had been on antiretroviral therapy for at least six months in any of the 17 AIDS centers in the Sughd Region.

### Study participants

In Tajikistan, all ART is delivered through public AIDS centers [21]. Our study included all people living with HIV registered in AIDS centers in the Sughd Region, who had been on ART for at least six months, had viral load test results at baseline and at six months, and had complete information for key variables in their health records at the AIDS centers. We excluded people under the age of 18, those who had not previously received ART, and those without a viral load test result from a test performed after having been on ART for at least 6 months.

### Data collection

We extracted data from the national electronic HIV case surveillance system, which contains epidemiological, clinical, and laboratory data. We also collected data from paper-based case report forms (containing demographic and epidemiological data) and paper-based outpatient registers (containing treatment and testing history). Data from the electronic database were cross-referenced with paper-based laboratory records, case report forms, and outpatient registers. Data were merged using a unique patient code. All personal identifying information was encoded for patient protection.

HIV viral load non-suppression was defined as a viral load of HIV above the detectable limit (≥ 1,000 copies/mL using HIV-1 RNA, quantitative, PCR assays) on their last viral load assessed. In Tajikistan, the standard of care for HIV treatment is for a viral load test every three months after ART initiation and every six months if the last two viral loads are below the detectable limit [21]. Time on ART was defined as the difference between the date of ART initiation and the date of the most recent viral load test. HIV disease stages I to IV were defined using the World Health Organization’s standard clinical definition of disease [22]. Behavioral data for self-reported HIV risk factors (modes of transmission and substance use history) were abstracted from patient case report forms completed by healthcare providers at the time of HIV diagnosis. A person was classified as a migrant if they moved away from their place of usual residence, across an international border, temporarily, after having been diagnosed with HIV [23].

### Statistical analysis

Data cleaning and analysis were performed in RStudio using R-4.4.0 (R Foundation for Statistical Computing, Vienna, Austria). Bivariate logistic regression analysis was conducted to identify potential clinical and socio-demographic characteristics associated with viral suppression. A multivariable Quasi-Poisson regression model was used to identify independent predictors of viral load non-suppression. Variables for the model were identified through the literature, assessment of plausible effects on the outcome, and potential modifiable relationships with other exposures. Statistically associated with viral suppression in the bivariable analysis were considered for the model. We assessed variables for correlation, interactions, and confounding, and retained the simplest model to avoid overfitting. Statistical significance was set at *p* < 0.05.

### Ethical considerations

Our study was conducted in accordance with the Declaration of Helsinki. Ethical approval for the study was received from the Ethics Review Committee of the Ministry of Health and Social Protection of the Population of the Republic of Tajikistan (No. 1–5/5282 dated 11.06.2024). This activity was also reviewed by the CDC, deemed non-research, and conducted in accordance with applicable federal laws and CDC policy (See e.g., 45 C.F.R. part 46.102(l)(2), 21 C.F.R. part 56; 42 U.S.C. § 241(d); 5 U.S.C. § 552a; 44 U.S.C. § 3501 et seq). Data were pulled retrospectively from medical registers and the national electronic HIV case surveillance system; therefore, patient consent was not obtained. Data were stored on a password-protected computer with limited access. All personally identifying information was removed from the analysis and replaced with a unique non-identifying code.

## Results

### Study selection

**Fig. 1:**
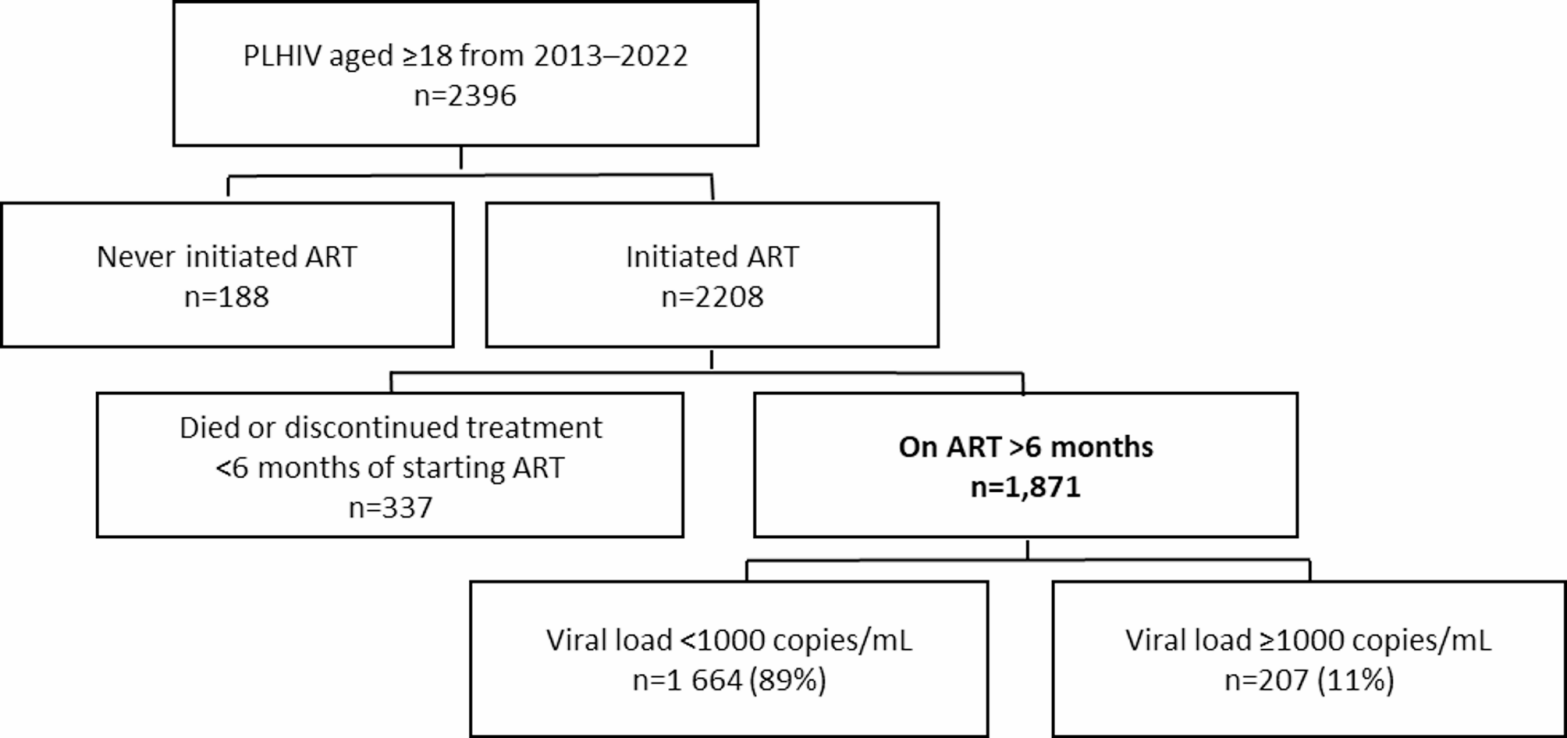
Participant recruitment diagram for people living with HIV registered at AIDS centers in Sughd Region, Republic of Tajikistan, from 2013 to 2022

Sughd region registered 2,396 people living with HIV aged 18 and older from 2013 to 2022. From these, we excluded 188 people who had never initiated ART and 337 who had died or discontinued treatment within 6 months of starting treatment (Fig. 1).

### Study characteristics

Of the 1,871 participants included in the study, 57% were male, and their median age was 31 years (range 18 to 74) (Table 1). Most (96%) had secondary education, 79% were unemployed, 73% were married, and 68% lived in rural areas. Migration after HIV diagnosis was documented for 38% of participants. Heterosexual transmission was the main self-reported mode of HIV infection for 89% of participants. Among participants, 6% had an alcohol use disorder, and 11% had a history of injection drug use.

**Table 1:** Socio-demographic and behavioral characteristics of people living with HIV who received ART in Sughd region, Tajikistan, 2013–2022

Characteristics	Participants *N*(column %)	Viral load non-suppression^1^ *n* (%)	*p*-value^2^
Overall	1871 (100%)	207 (11%)	
Sex			0.001
Female	802 (43%)	67 (8%)	
Male	1069 (57%)	140 (13%)	
Age			0.400
18–29	440 (24%)	49 (11%)	
30–39	722 (39%)	79 (11%)	
40–49	489 (26%)	48 (10%)	
50+	220 (12%)	31 (14%)	
Education			0.500
High	46 (3%)	4 (9%)	
Primary	23 (1%)	4 (17%)	
Secondary	1802 (96%)	199 (11%)	
Employment status			0.003
Other	65 (4%)	15 (23%)	
Unemployed	1480 (79%)	151 (10%)	
Employed	326 (17%)	41 (13%)	
Marital status			0.003
Married	1370 (73%)	133 (10%)	
Not married	164 (9%)	29 (18%)	
Divorced	337 (18%)	45 (13%)	
Place of residence			> 0.999
Urban	590 (32%)	66 (11%)	
Rural	1281 (68%)	141 (11%)	
Migrated after HIV diagnosis			0.002
Yes	717 (38%)	100 (14%)	
No	1,154 (62%)	107 (9%)	
Self-reported mode of transmission			< 0.001
Heterosexual	1666 (89%)	167 (10%)	
Parenteral (injecting drug use)	181 (10%)	35 (19%)	
Other or unknown	24 (1%)	5 (21%)	
Alcohol use disorder			< 0.001
Yes	121 (6%)	37 (19%)	
No	1750 (94%)	170 (10%)	
Ever used injection drugs			< 0.001
Yes	197 (11%)	37 (19%)	
No	1674 (89%)	170 (10%)	

^1^Most recent viral load test ≥ 1,000 copies/mL

^2^Chi-square (X^2^) *p*-value

Over half (58%) of participants initiated ART in 2013–2018 (Table 2). One-quarter (23%) were graded to be at WHO HIV stage III at diagnosis, and 9% at stage IV. The initial CD4 count was < 200 cells/mm^3^ in 14% of participants. More than half (55%) changed their ART regimen after having started ART, and 14% had stopped ART. The majority (94%) were on a TDF/3TC/DTG (tenofovir/lamivudine/dolutegravir) regimen at the time of the data collection. A history of tuberculosis was documented among 8% of participants, and 9% of participants had died.

**Table 2:** Clinical characteristics of people living with HIV who received ART in the Sughd region, Tajikistan, 2013–2022

Characteristics	Total participants *N* (column% %)	Viral load non-suppression^1^*n* (%)	*p*-value^2^
Year of ART initiation			< 0.001
2013–2018	1079 (58%)	150 (14%)	
2019–2022	792 (42%)	57 (7%)	
Years on ART ^3^			< 0.001
< 5 years	1,078 (58%)	138 (13%)	
5 + years	793 (42%)	69 (9%)	
HIV-stage at diagnosis			< 0.001
I	828 (44%)	72 (9%)	
II	445 (24%)	42 (9%)	
III	438 (23%)	57 (13%)	
IV	160 (9%)	36 (23%)	
CD4 test at diagnosis			< 0.001
< 200 cells/mm^3^	269 (14%)	19 (7%)	
≥ 200 cells/mm^3^	1602 (86%)	188 (12%)	
Ever changed ART regimen			< 0.001
Yes	1022 (55%)	45 (4%)	
No	588 (31%)	100 (17%)	
Stopped ART	261 (14%)	62 (24%)	
Current ART regimen			< 0.001
TDF/3TC/DTG	1759 (94%)	147 (8%)	
Other	112 (6%)	60 (54%)	
Any history of TB			< 0.001
Yes	153 (8%)	32 (21%)	
No	1718 (92%)	175 (10%)	
Died			< 0.001
Yes	164 (8.8%)	77 (47%)	
No	1,707 (91%)	130 (7.6%)	

*ART* Antiretroviral, *TDF/3TC/DTG* Tenofovir disoproxil fumarate, Lamivudine, Dolutegravir, *TB* tuberculosis

^1^Most recent viral load test ≥ 1,000 copies/mL

^2^Chi-square (X^2^) *p*-value

^3^Based on time from the initial date of ART inception and most recent viral load test

### Prevalence of viral load non-suppression

The proportion of people on ART with viral load non-suppression was 11% overall (Table 1). Prevalence of non-suppression was significantly greater for males vs. females (13% vs. 8%, respectively, *p* = 0.001). Non-suppression was higher among unmarried people (18%), those with primary education (17%), and those with other employment status (23%). People who had migrated after HIV diagnosis had a higher prevalence than non-migrants (14% vs. 9%, *p* = 0.002), and people who had ever injected drugs had a higher prevalence than non-injectors (19% vs. 10%, *p* < 0.001).

Prevalence of non-suppression was 14% for those who initiated ART in 2013–2018 (Table 2) compared to 7% for those who started ART in 2019–2022 (*p* < 0.001). It was higher for those on ART for less than five years compared to those on ART for five or more years (13% vs. 9%, *p* < 0.001). Prevalence differed significantly by HIV stage, and was 23% among those with HIV stage IV, 13% among those with stage III, and 9% among those with stage I or II (*p* < 0.001). Prevalence was 54% among people who were not on Tenofovir Disoproxil Fumarate, Lamivudine, and Dolutegravir (TDF/3TC/DTG) and 47% among people who had died.

### Factors associated with viral load non-suppression

In multivariable analysis (Table 3), higher risk of viral load non-suppression was observed among participants who were male migrants and male nonmigrants compared to female nonmigrants (adjusted relative risk and 95% confidence interval [aRR] = 1.61 [1.13–2.31] and aRR = 1.48 [1.03–2.14], respectively), never married vs. married (aRR = 1.56 [1.05–2.25]), who initiated ART in 2013–2018 vs. 2019–2022 (aRR = 1.92 [1.28–2.88]), who were not on a DTG-containing ART vs. DTG (aRR = 3.86 [2.63–5.69]), and who were on ART for < 5 years vs. longer (aRR = 1.56 [1.05–2.29]).

**Table 3:** Factors associated with viral load non-suppression^1^ among people living with HIV on treatment in the Sughd region (*N* = 1871), Tajikistan, 2013–2022

Characteristics	cRR	95% CI	*p*-value^2^	aRR	95% CI	*p*-value^2^
Migration by sex						
Female nonmigrant	Ref.			Ref.		
Male nonmigrant	1.66	1.16, 2.39	0.006	1.48	1.03, 2.14	0.035
Female migrant	1.79	1.09, 2.87	0.018	1.39	0.85, 2.24	0.200
Male migrant	2.00	1.42, 2.85	< 0.001	1.61	1.13, 2.31	0.008
Marital status						
Married	Ref.			Ref.		
Never married	1.82	1.23, 2.62	0.002	1.56	1.05, 2.25	0.021
Divorced	1.38	0.99, 1.88	0.050	1.29	0.92, 1.79	0.120
ART initiation period						
2013–2018	1.93	1.46, 2.59	< 0.001	1.92	1.28, 2.88	0.002
2019–2022	Ref.			Ref.		
Regimen						
TDR/3TC/DTG	Ref.			Ref.		
Other	6.41	4.80, 8.47	< 0.001	3.86	2.63, 5.69	< 0.001
Years on ART^3^						
< 5 years	1.47	1.34, 2.25	< 0.001	1.56	1.05, 2.29	0.026
5 + years	Ref.			Ref.		

*cRR* Crude relative risk, *CI* Confidence Interval, *aRR* Adjusted relative risk, *ART* Antiretroviral, *TDF/3TC/DTG* Tenofovir disoproxil fumarate, Lamivudine, Dolutegravir

^1^Most recent viral load test ≥ 1,000 copies/mL

^2^Quasi-Poisson regression

^3^Based on time from the initial date of ART inception and the most recent viral load test

## Discussion

We examined factors associated with viral load non-suppression among people living with HIV receiving ART from 2013 to 2022 in Sughd, Tajikistan. We found that one in ten (11%) people had unsuppressed viral loads. This does not meet the 5% target set by UNAIDS to end the HIV pandemic [13]. People diagnosed with advanced disease (a proxy for late diagnosis) had twice the risk of unsuppressed viral load compared to those with early-stage disease. Men, including those who migrated, had an increased risk of non-suppression compared to nonmigrant women.

Migrant populations are disproportionately affected by HIV globally [24]. Migrants with HIV face barriers to linkage and retention in HIV care due to their mobility, legal status, sociocultural influences, financial constraints, and stigmatization [25–27]. Tajikistan is a low-resource country where more than 40% of households reported at least one member working abroad [28]. It is estimated that over one million Tajiks migrate each year. Migration is driven by a lack of economic opportunities, and most migration is seasonal. In our study, two in every five people living with HIV migrated after their HIV diagnosis, and those who migrated had a 40% increased risk of non-suppression compared to those who resided permanently in Tajikistan. This finding is consistent with several other studies across countries that similarly confirmed that migrants have a higher risk of treatment failure [29, 30].

Russia is the destination for most Tajik labor migrants. Studies of male migrants in Russia showed that they had limited knowledge of HIV, lacked social support, and practiced unsafe risk behaviors that contributed to their vulnerability to HIV [31–33]. Additionally, migrants from Tajikistan in Russia have an increased risk of late HIV diagnosis, and for every year spent in Russia, the risk of late HIV diagnosis is estimated to increase by 4% [34]. Migrants with HIV cannot obtain residence permits per Russian legislation, and migrants with HIV living in Russia face the risk of deportation. Therefore, migrants with HIV face a difficult choice: loss of income by returning home for treatment or remaining in Russia without routine HIV care and treatment and risk deportation.

Non-suppression was associated with the year of treatment initiation and with the type of treatment regime. Specifically, people initiating ART before 2015 and those not on a DTG-containing treatment regimen had a higher prevalence of non-suppression than their counterparts. WHO first recommended the use of DTG-containing ART as first- or second-line treatment in 2018. That same year, Tajikistan adopted TDF/3TC/DTG as the first-line treatment for HIV. People newly diagnosed with HIV initiating ART were immediately initiated on DTG, and after 2018, people on other regimens were switched to the DTG-based regimen. The higher prevalence of viral suppression in our study among those taking a DTG-containing ART is consistent with other studies that demonstrate the increased viral suppression even with reduced adherence. However, some countries have started to show increasing rates of DTG resistance [35]. Monitoring of drug resistance in Tajikistan can help identify whether people on DTG who are not suppressed have resistance.

We found that non-suppression was greater for people who had recently initiated ART (< 5 years) compared to those who had been on ART for longer than 5 years. This finding was unexpected given that other studies have found that the risk of non-suppression increases over time [36]. However our findings can be explained by that one in three people in our study had advanced disease at the time of HIV diagnosis, and that one in 10 people who initiated ART died. People with advanced HIV disease at the time of diagnosis are less likely to achieve a suppressed viral load in 6 months and also have an increased risk of dying [37].

This study has some important limitations. First, because our study was a retrospective analysis of routinely collected programmatic data, we were limited to the variables available. For example, we did not have information on the frequency of migration, place of migration, or adherence to treatment. Therefore, it is not possible to determine whether non-suppression among migrants results from interruptions in treatment or other issues, such as drug resistance [38]. Second, study participants are only tracked from their time of HIV diagnosis and treatment initiation in Sughd. People who had a diagnosis and started treatment outside the country would appear as having a new diagnosis and newly initiating HIV in the system when, in fact, some migrants may have already been receiving care abroad. We, therefore, cannot know if virologic failure is due to new treatment failure or previous failure. Third, our results are likely biased by stigma, which is high in Tajikistan, particularly for key populations. As such, key population data are likely underreported. People living with HIV who are members of key populations, such as men who have sex with men, people who inject drugs, and sex workers, have a higher likelihood of experiencing non-suppressed viral load [39]. Understanding the specific barriers to achieving viral load suppression in these groups is essential, and this was not possible with our study design. Lastly, our study did not examine structural (e.g., clinic hours, drug dispensation options, informal fees for services) or social determinants (e.g., transportation challenges, economic hardships, stigma) that are known to be associated with ART non-suppression because these were not available in the routine data used for analysis [40, 41].

Despite these limitations, the present analysis provides valuable information on the potential determinants of viral load non-suppression among people living with HIV in the Sughd Region. Strategies for timely HIV detection and approaches that promote continuity of antiretroviral therapy might help reduce rates of viral load non-suppression [27, 42]. In particular, developing services to support migrant workers and men diagnosed with HIV could be pathways to improve viral load suppression in those high-risk subgroups.

## Conclusion

Our study, which aimed to identify factors associated with viral load non-suppression among people living with HIV receiving ART from 2013 to 2022 in Sughd Region, Tajikistan, found that being a male migrant, being unmarried, having advanced HIV disease at the time of diagnosis, being on a non-DTG containing regimen, and being on an ART for less than 5 years were associated with viral load non-suppression. These findings could be used to enhance efforts to engage people living with HIV in continuous ART treatment and to explore further the underlying reasons why these groups have an increased risk of non-suppression.

## Data Availability

The data that support the findings of this study are available on request from the corresponding author. The data are not publicly available due to privacy and ethical restrictions.

## Abbreviations

aRR: Adjusted relative risk
ART: Antiretroviral Therapy
CD4: Cluster of differentiation 4
CDC: United States Centers for Disease Control and Prevention
CI: Confidence intervals
DTG: Dolutegravir
HIV: Human Immunodeficiency Virus
RR: Relative risk
TB: Tuberculosis
TDF/3TC/DTG: Tenofovir Disoproxil Fumarate, Lamivudine, and Dolutegravir
WHO: World Health Organization
